# Downstream Gene Activation of the Receptor ALX by the Agonist Annexin A1

**DOI:** 10.1371/journal.pone.0012771

**Published:** 2010-09-17

**Authors:** Derek Renshaw, Trinidad Montero-Melendez, Jesmond Dalli, Ahmad Kamal, Vincenzo Brancaleone, Fulvio D'Acquisto, Giuseppe Cirino, Mauro Perretti

**Affiliations:** 1 William Harvey Research Institute, Barts and The London School of Medicine, Queen Mary University of London, London, United Kingdom; 2 Department of Experimental Pharmacology, School of Pharmacy, University of Naples, Naples, Italy; Fundação Oswaldo Cruz, Brazil

## Abstract

**Background:**

Our understanding of pro-resolution factors in determining the outcome of inflammation has recently gained ground, yet not many studies have investigated whether specific genes or patterns of genes, are modified by these mediators. Here, we have focussed on the glucocorticoid modulated pro-resolution factor annexin A1 (AnxA1), studying if its interaction with the ALX receptor would affect downstream genomic targets.

**Methodology/Principal Findings:**

Using microarray technology in ALX transfected HEK293 cells, we discovered an over-lapping, yet distinct gene activation profile for AnxA1 compared to its N-terminal mimetic peptide Ac2-26, which may be suggestive of unique downstream inflammatory outcomes for each substance. When the *up*-regulated genes were explored, consistently induced was the sphingosine phosphate phosphatase-2 gene (*SGPP2*), involved in regulation of the sphingosine 1 phosphate chemotactic system. Up-regulation of this gene, as well as JAG1 (and down-regulation of JAM3), was confirmed using real time PCR both with transfected HEK293 cells and human peripheral blood leukocytes. Furthermore, lymph nodes taken from AnxA1^null^ mice displayed lower *SGPP2* gene activity. Finally, connectivity map analysis for AnxA1 and peptide Ac2-26 indicated striking similarities with known anti-inflammatory therapeutics, glucocorticoids and aspirin-like compounds, as well as with histone deacetylase inhibitors.

**Conclusion/Significance:**

We believe these new data raise the profile of AnxA1 from being solely a short-term anti-inflammatory factor, to being a ‘trigger’ of the endogenous pro-resolution arsenal.

## Introduction

The concept of endogenous anti-inflammation has gained ground recently with various mediators being identified: they are actively involved in controlling and attenuating the potential over-reaction of the body's immune system, thereby affording a degree of protection for the host, assuring a strict time-dependence of the acute inflammatory response and promoting rapid regain of homeostasis [1], [2], [3], [4]. Among the group of pro-resolving endogenous anti-inflammatory mediators, glucocorticoids represent one of the main pathways. Released by the concerted action of hormones acting on the hypothalamus, pituitary and adrenal glands, glucocorticoids augment the cellular levels of a downstream anti-inflammatory mediator, the 37-kDa protein Annexin 1 (AnxA1, formerly named lipocortin-1). Blood-borne polymorphonuclear leukocyte (PMN) represents the first line of defence in innate immunity, as they are the first to rapidly extravasate to the site of inflammation. The function of early/non-genomic effects of AnxA1 on the PMN, in the context of the endogenous control against over-shooting of inflammation, are well characterised and include inhibition of PMN extravasation in models of acute [5] and chronic inflammation [6] as well as in experimental systemic inflammation [7] have been described.

Many of the cellular effects of AnxA1 are mediated by a specific G-protein-coupled 7-transmembrane receptor, termed ALX. This receptor is shared by another effector of endogenous anti-inflammation, the short-lived lipid lipoxin A_4_ [8], hence the acronym ALX for lipoxin A_4_ receptor. However, ALX is also structurally related to the human formyl-peptide receptor or FPR, hence sometimes it is referred to as FPR-like-1 or FPR2 [9]; [10]. Here, we will use the ALX classification, as this terminology is more relevant to the anti-inflammatory functions of this receptor. Human FPR is the classic receptor for the chemoattractant formyl-Met-Leu-Phe, whereas ALX displays ∼70% similarity at the nucleotide level, and binds several synthetic and natural ligands, examples of the latter ones being serum amyloid A, lipoxin A_4_ and AnxA1 [8], [11].

The biological anti-inflammatory actions of AnxA1 are by and large replicated by short peptides derived from the N-terminal sequence of the 346-aa long protein. For example, peptide Ac2-26 retains most of the anti-migratory actions of AnxA1 [12], [13] however, using artificial transfected cell systems, peptide Ac2-26 has been shown to activate human FPR [14], [15] as well as the third receptor of this family, termed FPR-like 2 [14]. We have recently transfected human FPR and ALX (the only two receptors of the group expressed by human PMN) in HEK293 cells finding that while AnxA1 displays selectivity for binding to ALX, the shorter and more flexible peptide Ac2-26, binds to both FPR and ALX with approximately equal affinity [16]. Analysis of the rapid post-receptor events indicated selective activation of the extracellular-regulated kinase 1 and 2 pathway, with no activation of other mitogen-activated protein kinase [16]. Finally, AnxA1 activation of ALX on the human PMN inhibited interaction with HUVEC monolayers as assessed with the flow chamber system.

The present study was undertaken to identify other effects downstream the AnxA1/ALX pathway, reasoning that delayed gene alteration might have implications in the control exerted by AnxA1 in complex and longer lasting inflammatory scenarios. In addition, a comparison between the genes altered by AnxA1 and its short N-terminal derived peptide Ac2-26 [17] was also made, supposing that a more rigid conformation, as in the full protein binding to ALX *vs.* a more flexible structure, as in the case of 24-aa long peptide Ac2-26, might incite distinct modes of activation of the receptor.

## Materials and Methods

### AnxA1 and Ac2-26 synthesis

Human AnxA1 cDNA was cloned into the expression vector pGEX-4T-327 and the protein expressed and purified (Scientific Proteins, Witterswil, Switzerland). AnxA1 was expressed as a fusion protein with major basic protein linked to the N-terminal and subsequently cleaved with TEV protease. Sodium dodecyl sulfate-polyacrylamide gel electrophoresis (SDS-PAGE) and western blot analysis showed that the recombinant AnxA1 was more than 98% pure, with an endotoxin contamination less than 40 U/mg as measured by the Limulus amebocyte chromogenic assay (Sigma, Poole UK). Peptide Ac2-26 (acetyl-AMVSEFLKQAWIENEEQEYVVQTVK; corresponding to residues 2-26 of human AnxA1) was synthesised by the Advance Biotechnology Centre (Imperial College School of Medicine, London, UK) using solid-phase stepwise synthesis. Purity was more than 90% as assessed by high-performance liquid chromatography (HPLC) and capillary electrophoresis (data supplied by the manufacturer).

### Receptor cloning and cell culture of stably transfected HEK293 cells

HEK293 cells were obtained form the European Collection of Cell Cultures (ECACC 85120602). ALX receptor was cloned in HEK293 cells and cultured as recently described [16]. Briefly, cloning the cDNA encoding the open reading frame of the receptor was obtained by PCR amplification of U937 cell cDNA library, using the forward primer, 5′-GCG CAA GCT TAT GGA AAC CAA CTT CTC CAC TCC TC, and reverse-purified and ligated in to pRc/CMV expression vector (Invitrogen, Paisley, UK), by sticky end ligation. The ALX construct was used to stably transfect HEK-293 cells using Fugene 6™ transfection reagent (Roche, Lewes, UK) according to manufacturer's instructions. In brief, 2 µg of plasmid DNA was used to transfect HEK-293 cells (2×10^5^ cells/well in 6 well plates) cultured in supplemented Eagle's minimum essential medium (EMEM). Neomycin (400 µg/ml; Invitrogen) was added to the medium to maintain selection.

### Cell Staining and Flow Cytometry

To detect membrane ALX, stably-transfected HEK293 cells were suspended in PBC buffer (0.15% BSA, 1 mM CaCl_2_ in PBS) and incubated with either primary monoclonal antibody for human ALX receptor (final concentration of 5 µg/ml; a kind gift from Dr Duncan Henderson (AstraZeneca, UK) or PBC alone. All samples were also incubated with human IgG at 320-µg/well (Sigma, UK) for 1 h at 4°C. Following washes with PBC, cells were incubated with rabbit anti-mouse FITC (STAR 9B, Serotec, UK) at 1∶40 dilution, for 45 minutes. Samples were washed and analysed by flow cytometry using a FAC-SCalibur (Becton Dickinson) cytometer. Data was acquired and analyzed using the CellQuest®. Mean fluorescence intensity was evaluated.

### Microarray Hybridization, Data Processing and Functional Analysis

The microarray analysis was performed on GeneChip® Human Genome U133 Plus 2.0 Arrays (Affymetrix, Inc. Santa Clara,CA), which include 54,675 probe sets that represent over 38,500 well-characterized human genes.

We used two replicates for each group: CMV (control group, HEK293 cells), ALX (HEK293 transfected with ALX receptor), Ac226 (HEK293 cells transfected with ALX receptor and treated with 10 µM Ac2-26 peptide) and AnxA1 (HEK293 cells transfected with ALX receptor and treated with 0.5 µM AnxA1 peptide).

All sample labelling, hybridization, staining and scanning procedures were carried out using Affymetrix standard protocols (www.affymetrix.com). Briefly, total RNA was purified 4 hours after treatment with the peptides, using Trizol (Invitrogen) method, according to manufacturer's instructions. To assess the quality of the RNA used for microarray experiments all samples were analysed using an Agilent Bioanalyser 2100. The RNA profiles for each sample were within the range recommended by the manufacturer. Then, cDNA was synthetized from total RNA and an *in vitro* transcription reaction was performed to obtain biotin-labelled cRNA, which was then fragmented and hybridized to the chips. After incubation, washing and staining steps, intensity signal for each probe set was measured.

Normalization was carried out using Bioconductor software (affyPLM package, RMA method). Normalized intensity values were used to obtain the fold change (FC) for each of the three groups studied (ALX alone, Ac2-26 and AnxA1) calculating the log_2_ of the ratio (average of the two replicates/average of the two control replicates). We also performed an exhaustive quality control study using Bioconductor (simpleAffy package), including visual inspection of the microarrays, % present genes, average background, scale factor, GAPDH 3′/5′ ratio and *spike in* controls. All of them showed normal values.

Data analysis and filtering were made with Spotfire DecisionSite v9.0. Genes with a FC more than a 50% compared to controls were considered differentially expressed. The functional analysis of differentially expressed genes (3120, 103 and 118 probes for ALX, Ac226 and AnxA1 groups respectively) was made using Panther Classification System v6.1 (Applied Biosystems).

Finally, according to MIAME recommendations and in order to ensure that all information is given in detail so results can be easily understood, reproduced and compared, all microarray data are publicly available in the Gene Expression Ommibus (GEO) database (GEO ID: GSE14807).

### Reverse Transcriptase (RT) PCR

RNA was extracted using Trizol solution according to the manufacturers instructions. ∼25 µg total RNA were treated with DNAse 1 (Ambion, UK) for 1 hr at 37°C. Total RNA for all experiments was normalised at 5 µg/reaction. cDNA was produced using SuperScript™ III (Invitrogen Ltd, Paisley,UK). PCR was performed using the following specific primers: hALX receptor: 5′-TAT TGC CAC CAA GAT CCA CA (forward, F), 5′-GA GGC AGC TGT TGA AGA AGG (reverse, R); hFPR: 5′-CAA CCC CAT GCT TTA CGT CT (F), 5′-T ATC CCT GAC CCC ATC CTC (R); hGAPDH: 5′-CAA ATT CCA TGG CAC CGT CA (F), 5′-GGA CTG GGT GTC GCT GTT GA (R). PCR amplification conditions were as follows, 94°C for 5 mins then 35 cycles of 94°C for 30 seconds, 59°C annealing for 30 seconds, 72°C extension for 30 seconds then 7 minutes at 72°C. Products were run immediately on a 1% agarose gel and photographed under ultra-violet light. Human primary neutrophil cDNA was used as a positive control for ALX receptor and FPR1 mRNA expression.

### Real-time-PCR using SYBR Green I Dye chemistry and data analysis

Samples were normalised to 40 ng of cDNA per well and loaded in triplicate for each gene. QuantiTect primer assays (Qiagen Ltd, UK) were used: hGAPDH (QT01192646); hRPL32 (QT00046088); hSGPP2 (QT00041832), hJAG1 (QT00031948), mJAM3 (QT00024997), mGAPDH (QT 0199388); mRPL32 (QT0131992) mSGPP2 (QT 01044204). Sample cDNA was obtained using SensIMix(dT) 2x mastermix and SYBR®Green 1 (Quantace Ltd, UK) using an ABI PRISM® 7900HT fast real time PCR system (ABI, Ca). PCR ramping protocols were standardised for all QuantiTect primer assay sets at 50°C for 2 min, 94°C for 5 min, followed by 40 cycles of 30 s at 94°C, 45 s at 55°C and 45 s at 72°C. GAPDH and RPL32 were used as endogenous controls. Data analysis was made using Relative Expression Software Tool (REST©).

### Animals

Animal work was performed by authorised scientists (holding a personal licence) according to UK Home Office regulations (Guidance on the Operation of Animals, Scientific Procedures, Act 1986) and complying with the directives of the European Union (project licence 70/5882 scrutinised by Queen Mary Ethics Committee first and then approved by the Home Office, on April 2004)". Male wild type (WT) Balb-c mice (20–25 g) were purchased from B&K (Hull, UK). AnxA1 null Balb-c mice (20–25) were backcrossed from the original mixed C57Bl6/129SV [18] for 10 generations. Five animals were housed per cage with 12 h light-dark cycle and controlled temperature. Animals were maintained on a standard chow pellet diet with tap water *ad libitum*.

### Isolation of primary human leukocytes

Experiments using healthy volunteers were approved by the local research ethics committee (05/Q0603/34 Barts and The London Research Ethics Commitee). Informed consent was provided according to the Declaration of Helsinki. Consent was verbal as per protocol approved, since dealing with healthy control blood. Each time samples were taken from healthy volunteers, a record of the amount of blood and date was written in a dedicated log book. Only blood from healthy volunteers was used for this study (no patient samples), which was used for leukocyte purification. Volunteers consented to allow the use of their white blood cells (via methods described below) for in vitro experiments. Blood was collected into 3.2% sodium citrate and diluted 1∶1 in RPMI-1640 (Sigma-Aldrich) before separation through a Histopaque 11191/10771 gradient. After PBMC/PMN isolation and washing, contaminating erythrocytes were removed by hypotonic lysis. PBMC/PMN's were resuspended at a concentration of 50×10^6^/mL in PBS supplemented with Ca^2+^ and Mg^2+^. PBMC/PMN cells were incubated at 37°C in a shaking water-bath for 4 hrs with and without human recombinant AnxA1 (500 nM). Following the incubation samples were centrifuged at 4000 g for 5 minutes and supernatant discarded. Samples were frozen at −80°C in Trizol until RNA purification.

## Results and Discussion

### Transfection of ALX receptor in HEK293 Cells

**Figure 1:**
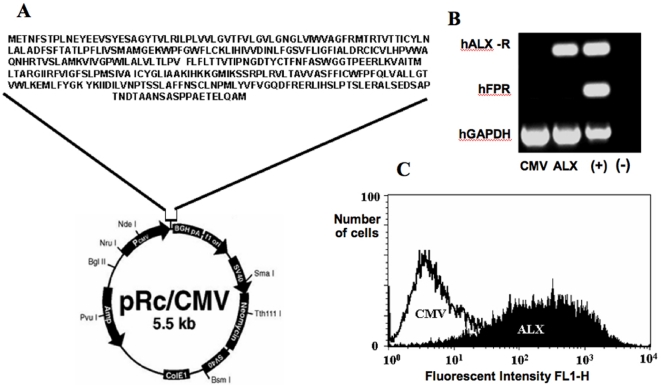
Transfection of ALX receptor into HEK293 cells. **A**) Generation of the HEK293 clones. pRc/CMV expression vector used to transfect ALX receptor into HEK293 cells to produce stable cell lines. Details of the full length protein sequence for the ALX receptor are also shown. **B**) Representative expression of FPR receptors by PCR visualised on a 1% agarose gel: CMV, empty vector transfected cells; ALX, annexin A1 receptor transfected cells; (+) human neutrophil cDNA positive control; (−) water replaced cDNA negative control. In all experiments results are representative of three separate experiments with similar results. **C**) Specific ALX receptor immunoreactive protein cell surface expression was determined using flow cytometry and compared to CMV stably transfected HEK293 cells.

HEK293 cells were chosen as a host cell for transfection experiments, as they lack endogenous FPR family receptors, including ALX receptor. The extent of ALX expression in stably transfected HEK293 cells was confirmed using specific primer sequences designed against the protein coding region of the human ALX receptor (Figure 1B). Control/empty vector stably transfected HEK293 cells demonstrate a lack of expression of either FPR or ALX receptor genes. Human neutrophil cDNA was used as a positive control. Expression of ALX receptor cell surface protein was demonstrated in unstimulated ALX stably transfected HEK293 cells compared to empty vector controls (CMV) using flow cytometry analysis (Figure 1C). There was a significant increase in positive fluorescent signal, in the ALX receptor transfected cells compared to CMV cells. No significant increase in fluorescent signal was observed when using an FPR specific antibody on either cell type (data not shown) demonstrating a lack of FPR receptor protein in these cells. Of equal importance, CMV or ALX-transfected cell incubation with 10 µM peptide Ac2-26 (4 h) did not yield any expression/release of endogenous AnxA1 (as assessed by Western blotting in cell-free supernatants), Lipoxin A4 or SAA (assessed by ELISA) (data not shown).

These results suggest that the ALX transfected HEK293 cells have both i) detectable cell surface expression in the ALX protein and ii) that this expression is specific to the cloning procedure, as CMV empty vector transfected cells show no immunoreactivity for the anti-ALX antibody. There was also no detectable release of AnxA1 protein from either transfected cell line (as measured by ELISA, data not shown), suggesting that there was no interference by endogenous AnxA1 protein in the transfected HEK293 cells in the subsequent microarray experiments.

### Identification of differentially expressed genes by AnxA1 and peptide Ac2-26

In order to obtain a global picture of gene expression changes associated with the activation of ALX receptor by the specific agonists AnxA1 and peptide Ac2-26, a whole-genome microarray study was carried out using GeneChip® Human Genome U133 Plus 2.0 (Affymetrix, Santa Clara, CA). HEK293 cells transfected with ALX receptor were treated with 0.5 µM AnxA1 or with 10 µM Ac2-26 for 4 hours; these concentrations were validated to produce rapid signalling responses (e.g. phospho-ERK) *via* ALX activation [16]. Cells containing the ALX receptor were used as controls. Also, a fourth group was included, consisting of HEK293 cells transfected with empty vector, which served as control to study the changes produced by the transfection of the receptor in these cells.

**Figure 2:**
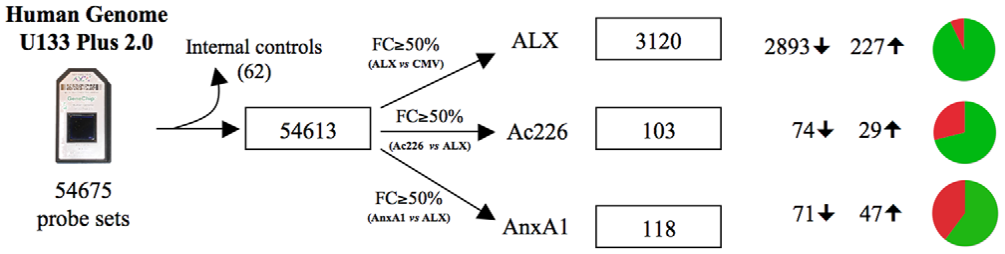
Filtering process and identification of differentially expressed probes in ALX, Ac226 and AnxA1 groups. Internal controls contained in the array were discarded. Then, probes showing a fold change (FC) less than 50% in expression for each group (ALX *vs.* CMV, Ac226 *vs.* ALX, AnxA1 *vs.* ALX) were selected as differentially expressed probes.

After an exhaustive quality control and normalization steps (see Material & Methods), Spotfire Decision Site software was used to identify differentially expressed genes. For each group (ALX, AnxA1 and Ac2-26), probes showing a fold change ≥50% compared to controls, were selected as differentially expressed. As shown in Figure 2, the transfection of the ALX receptor into HEK293 cells produced strong changes on gene expression: 3120 probes, of which 2893 were down-regulated and only 227 were up-regulated. This strong effect on gene expression could be due to the artificially over-expression of the receptor that is observed in these cells after the transfection. On the other hand, 118 and 103 probes were differentially expressed after treatment with AnxA1 and Ac2-26 peptides respectively, compared to control group (ALX). These genes are summarized in Supplementary Tables S1 and S2.

A succinct list of the interesting genes affected by both AnxA1 and Ac2-26 would include SGPP2 (sphingosine-1-phosphate phosphatase), an enzyme involved in the degradation of sphingosine-1-phosphate (S1P), a lipid mediator involved in several physiological processes including cell growth and survival, and leukocyte migration [19], [20]. S1P has also been shown to play a role in pathological conditions including vascular permeability, cancer and inflammation and recent reports show that SGPP2, one of the enzymes involved in its regulation is induced during the inflammatory process [21] as well as in experimental osteoarthritis [22].

Another gene of interest was JAM3 (junctional adhesion molecule 3, also known as JAMC), which was down-regulated by treatment with AnxA1. JAM3 is an adhesion molecule involved in transendothelial migration of leukocytes [23]. Recent data suggest potential roles for this protein in the pathogenesis of rheumatoid arthritis, being highly expressed in rheumatoid synovium. Moreover, targeting JAM3 significantly reduced the severity of experimental arthritic disease [24], [25]. We would propose that down-regulation of this gene by AnxA1 may contribute to its inhibitory properties in models of chronic inflammation. As an example, work of Morand and colleagues have highlighted the importance of this mediator in modulating inflammatory arthritis [6], [26]. Our new data indicate that, in inflammatory settings, expression of JAM-3 may be modulated upon AnxA1 administration (pharmacological studies) or AnxA1 deficiency (patho-physiological investigations).

**Figure 3:**
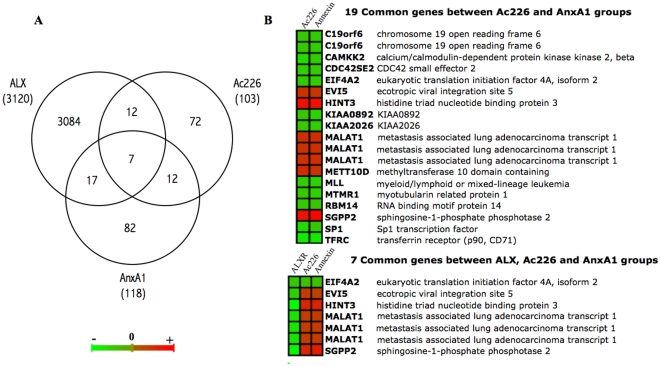
Common differentially expressed probes among ALX, AxnA1 and Ac2-26 groups. A) Venn diagram comparing the differentially expressed probes identified in each group (ALX, Ac226 and AnxA1). B) Expression of the 7 probes differentially expressed in all groups and the 19 probes differentially altered by the two peptides (Ac226 and AnxA1), obtained from the Venn diagram. Up-regulated probes are displayed in red and down-regulated in green.

Next, we used a distinct approach where we compared the three groups in order to find similarities and/or differences in gene expression changes that could be related to the mechanism of action of these peptides. The Venn diagram in Figure 3 reports the 7 common probes that are affected in the three groups. Surprisingly, only 19 probes were differentially expressed between AnxA1- and Ac2-26-treated cells. Some of these genes include the Sp1 transcription factor, SGPP2, EVI5 gene, involved in cell cycle regulation, the transferrin receptor (TFRC) or C19ORF6, also called membralin, which has been associated with ovarian carcinoma (Figure 3).

### Functional analysis of ALX, AnxA1 and Ac2-26 differentially expressed genes

**Figure 4:**
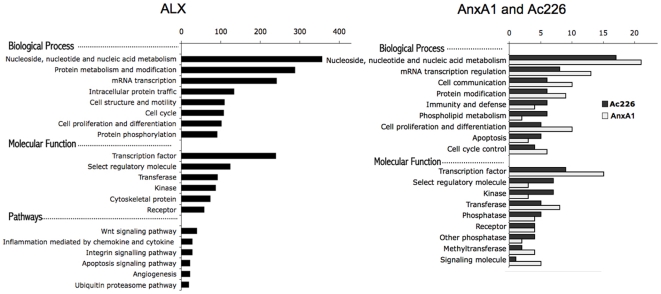
Functional analysis of differentially expressed genes. Representative categories of biological process, molecular functions and pathways are shown. Functional analysis of the 3120 differentially expressed probes in ALX group (*left*) and analysis of the 103 and 118 altered probes in Ac226 and AnxA1 groups, respectively (*right*). Panther Classification System was used for this analysis.

To further analyze the biological meaning of gene alteration detected in our experiments, we performed a functional study using Panther Classification System which classify genes according to biological process, molecular function and pathways. Figure 4 illustrates the most interesting categories involved in ALX transfected cells (left) and AnxA1 and Ac2-26 treated cells (right). Nucleic acid metabolism and protein metabolism and modification appear as the most affected processes in the ALX group. Other interesting categories include cell structure and motility, transcription factors as well as pathways related to immunity such as inflammation mediated by chemokine and cytokine and integrin signaling pathways (Figure 4).

As expected, ALX receptor (or FPR2) was up-regulated, showing a fold change of 1.55. Among genes related to immunity, we detected a significant down-regulation in IFNGR1 (interferon gamma receptor), IL6ST (interleukin 6 signal transducer) and several suppressors of cytokine signalling molecules (SOCS2, SOCS4, SOCS5 and SOCS6). In addition, several surface molecules, including CD109, CD164 and CD24, were down-regulated as well as chemokine (CXC motif) receptor 4, a chemotactic receptor with a critical role in tumor progression, and TIA1 (TIA cytotoxic granule-associated RNA binding protein), a protein with nucleolytic activity against cytotoxic lymphocyte target cells.

Regarding the treatment with AnxA1 or Ac2-26, they seem to affect predominantly the same functions, although some differences could also be observed. For example, AnxA1 altered a higher number of genes related to cell proliferation and differentiation, transcription factors and transferases, while peptide Ac2-26 affected more genes involved in immunity and defense, phospholipid metabolism and kinases, to name just a few. The most interesting genes emerged in our study are those related to immune defense. Some of the genes in this category include SP1, a transcription factor involved in the immune response, which is down-regulated by both peptides.

**Figure 5:**
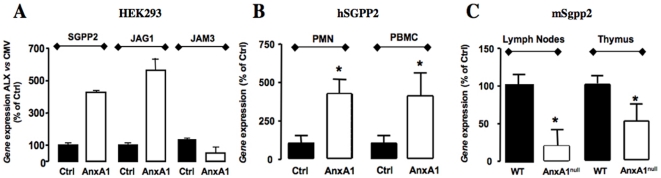
Real time-PCR validation. A) Gene expression of SGPP2, JAG1 and JAM3 in ALX-HEK293 transfected cells compared to empty vector-transfected cells (CMV group). B) Gene expression of SGPP2 in human peripheral mononuclear cells (PBMC) and polymorphonuclear cells (PMN), under control conditions (Ctrl) or after 4 h incubation with 0.5 µM AnxA1 (AnxA1). C) mSGPP2 gene expression in lymph nodes and thymuses of wild type (WT) and AnxA1^null^ mice. (*p<0.05, t-Test compared to controls (A,B) or wild type (C)).

Another striking finding was the up-regulation of JAG1 consequent to AnxA1. Microarray data were confirmed by real time PCR (Figure 5A). The protein encoded by this gene is the ligand for the receptor Notch 1, which plays an important role during T-cell development. This is in line with recent observations of our group [27] where AnxA1 modulates not only the innate system, but also T-cell functions. Furthermore, it has been reported that administration of anti-JAG1 antibody exacerbates experimental autoimmune encephalomyelitis [28], suggesting a potential role of this gene as a therapeutic target for inflammatory conditions. We believe these novel findings will feed into future studies aiming at clarify the complex and positive actions of AnxA1 on T cells and other immune responses [13], [29].

### Real Time-PCR validation of selected genes

In order to confirm the gene expression changes obtained in the microarray experiment, we performed real time-PCRs using SYBR Green I Dye chemistry. We focused here on three genes: SGPP2, as it controls the trafficking of lymphocytes in the thymus and lymphoid organs [19], JAG1, which is related to T cell function and could be related to the role of AnxA1 in the adaptive immune system [13], [29], and JAM3, as previously mentioned. Quantitative PCR experiments confirmed that the altered gene expression of these genes by AnxA1 was genuine (Figure 5A). Although HEK293 cells lack receptors for AnxA1, these experiments were carried out using empty vector-transfected cells (CMV) treated with AnxA1 as controls, showing therefore that the effect of the protein is genuinely due to interaction with ALX, and not the result of non-specific interaction with other receptors present on the plasma membrane of HEK293 cells.

Next, we focused on SGPP2 gene to perform further analyses. Thus we could confirm its over-expression in PBMCs and PMNs isolated from healthy human donors after incubation with 0.5 µM AnxA1 for 4 hours. Figure 5B shows a significant (*p*<0.01, n = 6) increase in SGPP2 expression in both type of cells, suggesting that AnxA1 can up-regulate SGPP2 expression not only in an artificial system (e.g. transfected HEK293 cells which over-express ALX), but also in primary human leukocytes that naturally express the receptor.

### SGPP2 expression in AnxA1^null^ mice

Although the function of AnxA1 has been mainly described in terms of the innate immune system, recent data suggest a role for AnxA1 in the adaptive immune system and the determination of Th1/Th2 phenotypes of T cells [13], [27]. According to this recent line of research, and supported by our novel finding of the up-regulation of SGPP2 enzyme by AnxA1, we decided to investigate the relationship between AnxA1, T cells and SGPP2. Thus, we compared the expression levels of SGPP2 in peripheral lymph node and thymus tissues from AnxA1^null^ and wild type (WT) animals by real time-PCR.

Figure 5C shows a significant decrease in SGPP2 expression in both tissues, when they were harvested from AnxA1^null^ mice, strongly suggesting a key regulatory function for AnxA1 on constitutive SGPP2 gene expression. There is positive regulation upon AnxA1 (or peptide Ac2-26) addition, conveyed by ALX activation, and there is also negative regulation in tonic absence of this protein, as observed in AnxA1^null^ mice.

### Relationship of AnxA1 and Ac2-26 to other compounds using a gene-expression based screening approach

The Connectivity Map is a repository of the gene expression profiles of cultured human cells exposed to small bioactive molecules. It contains more than 7000 gene expression signatures representing 1309 different compounds. This map represents a tool for the large-scale discovery of connections among small molecules, genes and diseases [30], [31]. This tool was used to reveal potential expected, and unexpected, connections between AnxA1 and Ac2-26 peptides and other chemical compounds, which could provide new information about their mechanism(s) of action. This approach has been recently applied with success to reveal the HSP90 inhibitory activities shared by a natural antioxidant molecule celastrol and the antimalarial agent gedunin [32]. Furthermore, Wei *et al* used the Connectivity Map to demonstrate that rapamycin modulates glucocorticoid resistance [33]. Therefore, we queried the Connectivity Map using the AnxA1 and Ac2-26 expression signatures obtained in our microarray analysis. The result is a ranking of all compounds included in the map, ordered by their similarity with our data: molecules with an expression profile similar to the expression profile of interest (e.g. AnxA1) will be found at the top of the ranking (positive score), while drugs displaying an opposite expression profile (negative score) will be located at the bottom of the list.

**Figure 6:**
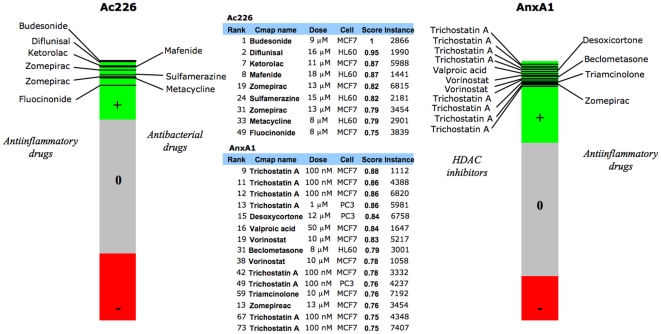
Connectivity Map results. Connectivity Map analysis of differentially expressed genes by 10 uM Ac2-26 peptide (103 genes) -*left*- and 0.5 uM AnxA1 (118 genes) -*right*-, in HEK293 cells transfected with the receptor ALX. Bar-views are constructed from more than 7,000 horizontal lines, each representing an individual treatment instance, ordered by their corresponding connectivity scores (+1, top; -1, bottom). Drugs with positive score are represented in green, while those with negative connectivity score are displayed in red. Only compounds with a positive score ≥0.75 are reported. A selection of these compounds is summarized in the table (middle).

Figure 6 summarises our analyses for AnxA1 and peptide Ac2-26. It was re-assuring that the Connectivity Map found several connections between our peptides and known anti-inflammatory drugs. For example, peptide Ac2-26 displayed similarities in gene expression patterns with glucocorticoids such as budesonide (score of 1) and fluocinonide (score of 0.75), as well as with several NSAIDs including diflunisal, ketorolac and zomepirac (scores of 0.95, 0.87 and 0.82, respectively). In a similar fashion, several anti-inflammatory drugs showed positive connectivity score with AnxA1 (see Figure 6). With respect to novel *connections*, it was interesting to note a high positive score for peptide Ac2-26 and antibacterial drugs as well as, perhaps more importantly for AnxA1, with histone deacetylase inhibitors (HDACIs).

In the first case, one hypothesis that can be formulated from this relation refers to the potential role of peptide Ac2-26 as anti-bacterial, as occurs with many other peptides, including melanocortin peptides [34], [35]. In order to test this hypothesis, we performed a minimum inhibitory concentration assay using several microorganisms (*E. coli, P. aeruginosa* and MRSA), following incubation with a concentration-range of peptide Ac2-26, but no significant effect was observed (data not shown). However, a different hypothesis might also emerge, inasmuch as antibacterial compounds might produce anti-inflammatory effects similar to those of peptide Ac2-26. Further studies will test this hypothesis, though it is worth noting that in the case of some antibiotics, such anti-inflammatory properties have already been shown (e.g. for tetracyclines see [36]). We identified metacycline connected to peptide Ac2-26 (score of 0.79; Figure 6). Furthermore, sulfasalazine is a sulfonamide that, in addition to its use as an antibiotic, is commonly used for the treatment of rheumatoid arthritis and inflammatory bowel disease patients. Our analysis highlighted two sulfonamides, sulfamerazine (score of 0.82) and mafenide (score of 0.87), suggesting that it could be worthwhile pursuing this type of compound as potential anti-inflammatory drugs.

The results obtained from AnxA1 were also very striking. We observed a strong, and unexpected, connection between AnxA1 and HDACIs, with several hits for Trichostatin A (scores ranging from 0.88 to 0.75). These compounds have already been successfully used as anti-inflammatory drugs in several experimental models, including rheumatoid arthritis [37], colitis [38] and traumatic brain injury [39].

HDACIs have been extensively studied as anticancer drugs due to their ability to induce apoptosis but the mechanism of action is not completely clear. Two recent reports suggest that the mechanism of action of HDACI-induced apoptosis might be linked to up-regulation of the pro-apoptotic protein AnxA1 [40]. On the other hand, AnxA1 augments leukocyte apoptosis and favours the removal of apoptotic cells by phagocytes (efferocytosis) [41], [42]. According to this and the novel connection between AnxA1 and HDACIs found in our results, we can hypothesise that - in line with anti-cancer activities [43] - the anti-inflammatory mechanism of action of HDACIs (still a matter of debate) could entail up-regulation of one of the effectors of endogenous anti-inflammatory processes, the protein AnxA1.

The Connectivity Map analysis also demonstrated similar profiles of gene expression for AnxA1 and two widely used glucocorticoids, beclomethasone and triamcinolone. Therefore, our genomic analysis validated long-dated experiments that have demonstrated the intermediate role for AnxA1 in specific anti-inflammatory activities of glucocorticoids (summarised in [44]).

In conclusion, this study reports the novel observation that AnxA1 is a regulator of SGPP2 in an ALX-dependent fashion. This gene was not only up-regulated by ALX-HEK293 cells but also in primary cells, i.e. human PMN and PBMCs. Conversely, lower SGPP2 expression levels were measured in tissues taken from AnxA1^null^ mice. Altogether, these data indicated potentially strong and novel links between the AnxA1 pathway and S1P biology, paving the way for further functional investigations.

Another up-regulated gene, validated by real time PCR, was JAG1, pointing to a regulatory role of AnxA1 on T cells by means of sustained alteration of genes central to their development and differentiation. The down-regulation of JAM3 is also interesting as it is involved in leukocyte migration [23], a process in which AnxA1 have already been implicated. For instance, AnxA1^null^ mice display a higher degree of leukocyte trans-migration across post-capillary venules in response to a variety of stimuli [45], which could be consequent to higher JAM3 expression hence function to sustain cell migration. Future studies will challenge this hypothesis.

An interesting observation was the partial overlap between the AnxA1 and peptide Ac2-26 signature. Peptide Ac2-26 can mimic many of the anti-inflammatory properties of the parent protein, but it is clear that there may be downstream events which are specific for AnxA1. On the other, we have previously reported a distinct functional behavour of peptide Ac2-26 compared to AnxA1 in human neutrophils [16]; this was explained with the ability of this peptide to bind many receptors of the FPR family [14]. However, more relevant here since the HEK cells were negative for FPR1, is the possibility that a part, possibly small, of the effects of peptide Ac2-26 might be due to actions independent from activation of any member of the FPR family [10].

Important from a pharmacological (and drug development) perspective, we have shown here that microarray studies can be used for more than merely identifying specific genes which are altered Gene expression signature-based approaches can be very instrumental for formulating hypotheses about novel mechanisms of actions or new potential activities of known drugs. We report a similar signature, partly expected, between AnxA1 and glucocorticoids and the novel association between AnxA1 and HDACIs; the latter, again, could act as a springboard for a comprehensive assessment of the novel biological properties of all these compounds

## Supporting Information

Table S1 Ac2-26 affected genes. Differentially expressed genes (FC greater than or equal to 50%) in HEK293 cells transfected with ALX receptor and treated with 0.5 uM Ac2-26 peptide during 4 hours.
(0.04 MB PDF)

Table S2 AnxA1 affected genes. Differentially expressed genes (FC greater than or equal to 50%) in HEK293 cells transfected with ALX receptor and treated with 10 uM AnxA1 peptide during 4 hours.
(0.04 MB PDF)
