# Supplementary material for: Downstream Gene Activation of the Receptor ALX by the Agonist Annexin A1
Source: PLoS One. 2010 Sep 17;5(9):e12771. doi: 10.1371/journal.pone.0012771 (PMC2941452; doi:10.1371/journal.pone.0012771)
Supplement: Table S1 — Ac2-26 affected genes. Differentially expressed genes (FC greater than or equal to 50%) in HEK293 cells transfected with ALX receptor and treated with 0.5 uM Ac2-26 peptide during 4 hours. (0.04 MB PDF) [file pone.0012771.s001.pdf]

Supplementary Table S1: 103 Differentially expressed genes in Ac226 group

Probe Set ID	FC	Symbol	Gene Title	Representative Public ID	Chromosomal Location
224568_x_at	-1.25	**MALAT1**	metastasis associated lung adenocarcinoma transcript 1 (non-protein coding)	AW005982	chr11q13.1
237215_s_at	-1.07	**TFRC**	transferrin receptor (p90, CD71)	N76327	chr3q29
1553749_at	-0.94	**FAM76B**	family with sequence similarity 76, member B	NM_144664	chr11q21
213986_s_at	-0.89	**C19orf6**	chromosome 19 open reading frame 6	AI805266	chr19p13.3
228742_at	-0.87	**---**	---	AA745978	---
1553685_s_at	-0.85	**SP1**	Sp1 transcription factor	NM_138473	chr12q13.1
210787_s_at	-0.83	**CAMKK2**	calcium/calmodulin-dependent protein kinase kinase 2, beta	AF140507	chr12q24.2
211027_s_at	-0.80	**IKBKB**	inhibitor of kappa light polypeptide gene enhancer in B-cells, kinase beta	BC006231	chr8p11.2
1553321_a_at	-0.79	**SULT1C4**	sulfotransferase family, cytosolic, 1C, member 4	NM_006588	chr2q11.1-q11.2
215357_s_at	-0.79	**POLDIP3**	polymerase (DNA-directed), delta interacting protein 3	Z93241	chr22q13.2
1560689_s_at	-0.78	**AKT2**	V-akt murine thymoma viral oncogene homolog 2	AK098099	chr19q13.1-q13.2
224534_at	-0.75	**KREMEN1**	kringle containing transmembrane protein 1	AB059618	chr22q12.1
205439_at	-0.73	**GSTT2**	glutathione S-transferase theta 2	NM_000854	chr22q11.2|22q11.23
209198_s_at	-0.72	**SYT11**	synaptotagmin XI	BC004291	chr1q21.2
217310_s_at	-0.71	**FOXJ3**	forkhead box J3	AK027075	chr1pter-q31.3
211205_x_at	-0.71	**PIP5K1A**	phosphatidylinositol-4-phosphate 5-kinase, type I, alpha	U78577	chr1q22-q24
223578_x_at	-0.69	**MALAT1**	metastasis associated lung adenocarcinoma transcript 1 (non-protein coding)	AF113016	chr11q13.1
217924_at	-0.69	**C6orf106**	chromosome 6 open reading frame 106	AL523965	chr6p21.31
216205_s_at	-0.69	**MFN2**	mitofusin 2	AK021947	chr1p36.22
214336_s_at	-0.69	**COPA**	coatomer protein complex, subunit alpha	AI621079	chr1q23-q25
216624_s_at	-0.68	**MLL**	myeloid/lymphoid or mixed-lineage leukemia (trithorax homolog, Drosophila)	Z69744	chr11q23
209401_s_at	-0.68	**SLC12A4**	solute carrier family 12 (potassium/chloride transporters), member 4	AI817690	chr16q22.1
211507_s_at	-0.68	**MTMR3**	myotubularin related protein 3	AF233437	chr22q12.2
213482_at	-0.68	**DOCK3**	dedicator of cytokinesis 3	BF593175	chr3p21.31
215581_s_at	-0.67	**MCM3AP**	minichromosome maintenance complex component 3 associated protein	AK022303	chr21q22.3
1558445_at	-0.67	**---**	---	AW827204	---
203953_s_at	-0.67	**CLDN3**	claudin 3	BE791251	chr7q11.23
1552612_at	-0.67	**CDC42SE2**	CDC42 small effector 2	NM_020240	chr5q31.1
207332_s_at	-0.67	**TFRC**	transferrin receptor (p90, CD71)	NM_003234	chr3q29
1558214_s_at	-0.66	**CTNNA1**	catenin (cadherin-associated protein), alpha 1, 102kDa	BG330076	chr5q31
223789_s_at	-0.66	**GTPBP2**	GTP binding protein 2	AF116627	chr6p21-p12
234939_s_at	-0.66	**PHF12**	PHD finger protein 12	AL161953	chr17q11.2
205076_s_at	-0.66	**MTMR11**	myotubularin related protein 11	NM_006697	chr1q12-q21
206184_at	-0.65	**CRKL**	v-crk sarcoma virus CT10 oncogene homolog (avian)-like	NM_005207	chr22q11|22q11.21
215064_at	-0.65	**SC5DL**	sterol-C5-desaturase (ERG3 delta-5-desaturase homolog, S. cerevisiae)-like	AK027246	chr11q23.3
223940_x_at	-0.65	**MALAT1**	metastasis associated lung adenocarcinoma transcript 1 (non-protein coding)	AF132202	chr11q13.1
231169_at	-0.65	**---**	---	N29877	---
241995_at	-0.64	**DGUOK**	deoxyguanosine kinase	BF511285	chr2p13

1555996_s_at	-0.64	**EIF4A2**	eukaryotic translation initiation factor 4A, isoform 2	AI332397	chr3q28
212325_at	-0.64	**LIMCH1**	LIM and calponin homology domains 1	AK027231	chr4p13
229743_at	-0.64	**ZNF438**	zinc finger protein 438	AI886666	chr10p11.23
1559421_at	-0.63	**---**	---	AV697037	---
242042_s_at	-0.63	**LOC401022**	hypothetical LOC401022	AA406052	chr2q31.2
202476_s_at	-0.63	**TUBGCP2**	tubulin, gamma complex associated protein 2	BF002130	chr10q26.3
1555639_a_at	-0.63	**RBM14**	RNA binding motif protein 14	AF315633	chr11q13.1
1557616_at	-0.62	**ZNF496**	zinc finger protein 496	AK098030	chr1q44
1569296_a_at	-0.62	**LOC493754**	Hypothetical LOC441245	BC029469	chr7q11.21
240528_s_at	-0.62	**EXOC4**	exocyst complex component 4	AI964022	chr7q31
228667_at	-0.62	**AGPAT4**	1-acylglycerol-3-phosphate O-acyltransferase 4	AI733330	chr6q26
217598_at	-0.62	**---**	---	BG236351	---
1553369_at	-0.62	**FAM129C**	family with sequence similarity 129, member C	NM_173544	chr19p13.11
238490_at	-0.62	**KIAA2026**	KIAA2026	BG109896	chr9p24.1
212574_x_at	-0.61	**C19orf6**	chromosome 19 open reading frame 6	AC004528	chr19p13.3
236121_at	-0.61	**OR51E2**	olfactory receptor, family 51, subfamily E, member 2	AI805082	chr11p15
244885_at	-0.61	**---**	---	AI016316	---
244619_at	-0.60	**LOC646626**	hypothetical LOC646626	AA521418	chr1p22.3
1558515_at	-0.60	**---**	---	AK057701	---
203890_s_at	-0.60	**DAPK3**	death-associated protein kinase 3	BF686824	chr19p13.3
219234_x_at	-0.60	**SCRN3**	secernin 3	NM_024583	chr2q31.1
214975_s_at	-0.60	**MTMR1**	myotubularin related protein 1	AK001816	chrXq28
216450_x_at	-0.60	**HSP90B1**	heat shock protein 90kDa beta (Grp94), member 1	AK025862	chr12q24.2-q24.3
228634_s_at	-0.60	**CSDA**	Cold shock domain protein A	BF195718	chr12p13.1
237020_at	-0.60	**TMEM146**	transmembrane protein 146	AI203106	chr19p13.3
223143_s_at	-0.59	**AKIRIN2**	akirin 2	AI742378	chr6q15
239432_at	-0.59	**FLJ31306**	hypothetical protein FLJ31306	AV729086	chr14q23.1
215672_s_at	-0.59	**AHCYL2**	S-adenosylhomocysteine hydrolase-like 2	AK025372	chr7q32.1
1570078_a_at	-0.59	**DOCK5**	dedicator of cytokinesis 5	BC011877	chr8p21.2
242743_at	-0.59	**IL4R**	Interleukin 4 receptor	AA767714	chr16p12.1-p11.2
222311_s_at	-0.59	**SFRS15**	splicing factor, arginine/serine-rich 15	AA648521	chr21q22.1
222385_x_at	-0.59	**SEC61A1**	Sec61 alpha 1 subunit (S. cerevisiae)	AF346602	chr3q21.3
216926_s_at	-0.59	**KIAA0892**	KIAA0892	AC003030	chr19p13.11
226530_at	-0.59	**BMF**	Bcl2 modifying factor	AK024472	chr15q14
230722_at	-0.59	**BNC2**	basonuclin 2	AI377043	chr9p22.3-p22.2
212647_at	-0.59	**RRAS**	related RAS viral (r-ras) oncogene homolog	NM_006270	chr19q13.3-qter
219303_at	0.59	**RNF219**	ring finger protein 219	NM_024546	chr13q31.1
205059_s_at	0.59	**IDUA**	iduronidase, alpha-L-	NM_000203	chr4p16.3
213176_s_at	0.60	**LTBP4**	latent transforming growth factor beta binding protein 4	AI910869	chr19q13.1-q13.2
242691_at	0.60	**---**	---	AA829017	---
1558605_at	0.61	**---**	---	BC040287	---

206177_s_at	0.61	**ARG1**	arginase, liver	NM_000045	chr6q23
222803_at	0.61	**PRTFDC1**	phosphoribosyl transferase domain containing 1	AI871620	chr10p12.1
225711_at	0.61	**ARL6IP6**	ADP-ribosylation-like factor 6 interacting protein 6	AA654338	chr2q23.3
228480_at	0.61	**VAPA**	VAMP (vesicle-associated membrane protein)-associated protein A, 33kDa	AW296039	chr18p11.22
226444_at	0.61	**---**	---	AI700476	---
228702_at	0.62	**FLJ43663**	hypothetical LOC378805	AL569506	chr7q32.3
213575_at	0.62	**TRA2A**	transformer-2 alpha	AA831170	chr7p15.3
1569713_at	0.63	**---**	---	BC009800	---
238004_at	0.64	**PGBD2**	piggyBac transposable element derived 2	BF344017	chr1q44
210858_x_at	0.64	**ATM**	ataxia telangiectasia mutated	U26455	chr11q22-q23
226537_at	0.64	**HINT3**	histidine triad nucleotide binding protein 3	AW418666	chr6q22.32
226982_at	0.64	**ELL2**	elongation factor, RNA polymerase II, 2	AI745624	chr5q15
226675_s_at	0.67	**MALAT1**	metastasis associated lung adenocarcinoma transcript 1 (non-protein coding)	W80468	chr11q13.1
228919_at	0.67	**---**	---	AA601031	---
208297_s_at	0.68	**EVI5**	ecotropic viral integration site 5	NM_005665	chr1p22.1
200630_x_at	0.69	**SET**	SET nuclear oncogene	AV702810	chr9q34
232465_at	0.69	**---**	---	AK021749	---
243372_at	0.69	**HSPD1**	heat shock 60kDa protein 1 (chaperonin)	AW674195	chr12q13.2
238931_at	0.75	**METT10D**	methyltransferase 10 domain containing	AL045793	chr17p13.3
243921_at	0.79	**---**	---	AW340093	---
226106_at	0.80	**RNF141**	ring finger protein 141	AI307808	chr11p15.4
236428_at	0.82	**---**	---	D59900	---
244780_at	1.04	**SGPP2**	sphingosine-1-phosphate phosphotase 2	AI800110	chr2q36.1
228697_at	1.09	**HINT3**	histidine triad nucleotide binding protein 3	AW731710	chr6q22.32
