# Supplementary material for: Downstream Gene Activation of the Receptor ALX by the Agonist Annexin A1
Source: PLoS One. 2010 Sep 17;5(9):e12771. doi: 10.1371/journal.pone.0012771 (PMC2941452; doi:10.1371/journal.pone.0012771)
Supplement: Table S2 — AnxA1 affected genes. Differentially expressed genes (FC greater than or equal to 50%) in HEK293 cells transfected with ALX receptor and treated with 10 uM AnxA1 peptide during 4 hours. (0.04 MB PDF) [file pone.0012771.s002.pdf]

Supplementary Table S2: 118 Differentially expressed genes in AnxA1 group

Probe Set ID	FC	Gene	Gene Title	Representative Public ID	Chromosomal Location
224568_x_at	-1.13	**MALAT1**	metastasis associated lung adenocarcinoma transcript 1 (non-protein coding)	AW005982	chr11q13.1
237215_s_at	-1.06	**TFRC**	transferrin receptor (p90, CD71)	N76327	chr3q29
213986_s_at	-0.99	**C19orf6**	chromosome 19 open reading frame 6	AI805266	chr19p13.3
231576_at	-0.94	**---**	---	AA829940	---
227260_at	-0.92	**ANKRD10**	Ankyrin repeat domain 10	AV724266	chr13q34
224589_at	-0.83	**XIST**	X (inactive)-specific transcript (non-protein coding)	BF223193	chrXq13.2
207781_s_at	-0.81	**ZNF711**	zinc finger protein 711	NM_021998	chrXq21.1-q21.2
204506_at	-0.78	**PPP3R1**	protein phosphatase 3 (formerly 2B), regulatory subunit B, alpha isoform	AL544951	chr2p14
216926_s_at	-0.76	**KIAA0892**	KIAA0892	AC003030	chr19p13.11
216624_s_at	-0.75	**MLL**	myeloid/lymphoid or mixed-lineage leukemia (trithorax homolog, Drosophila)	Z69744	chr11q23
231721_at	-0.74	**JAM3**	junctional adhesion molecule 3	AF356518	chr11q25
214975_s_at	-0.73	**MTMR1**	myotubularin related protein 1	AK001816	chrXq28
219627_at	-0.73	**ZNF767**	zinc finger family member 767	NM_024910	chr7q36.1
206241_at	-0.72	**KPNA5**	karyopherin alpha 5 (importin alpha 6)	NM_002269	chr6q22.2
211708_s_at	-0.71	**SCD**	stearoyl-CoA desaturase (delta-9-desaturase)	BC005807	chr10q24.31
223940_x_at	-0.71	**MALAT1**	metastasis associated lung adenocarcinoma transcript 1 (non-protein coding)	AF132202	chr11q13.1
228157_at	-0.70	**ZNF207**	zinc finger protein 207	AI125646	chr17q11.2
208097_s_at	-0.69	**TXNDC1**	thioredoxin domain containing 1	NM_030755	chr14q22.1
238490_at	-0.69	**KIAA2026**	KIAA2026	BG109896	chr9p24.1
1555266_a_at	-0.69	**ASXL2**	additional sex combs like 2 (Drosophila)	BC042999	chr2p24.1
1555996_s_at	-0.69	**EIF4A2**	eukaryotic translation initiation factor 4A, isoform 2	AI332397	chr3q28
200769_s_at	-0.68	**MAT2A**	methionine adenosyltransferase II, alpha	NM_005911	chr2p11.2
205321_at	-0.67	**EIF2S3**	eukaryotic translation initiation factor 2, subunit 3 gamma, 52kDa	NM_001415	chrXp22.2-p22.1
220342_x_at	-0.67	**EDEM3**	ER degradation enhancer, mannosidase alpha-like 3	NM_017992	chr1q24-q25
241820_at	-0.67	**RIF1**	RAP1 interacting factor homolog (yeast)	BF666241	chr2q23.3
209535_s_at	-0.67	**---**	---	AF127481	---
235446_at	-0.66	**---**	---	AW856618	---
200672_x_at	-0.66	**SPTBN1**	spectrin, beta, non-erythrocytic 1	NM_003128	chr2p21
242558_at	-0.66	**---**	---	AW362945	---
1555515_a_at	-0.66	**C1orf2**	chromosome 1 open reading frame 2	BC008854	chr1q21
210787_s_at	-0.65	**CAMKK2**	calcium/calmodulin-dependent protein kinase kinase 2, beta	AF140507	chr12q24.2
227245_at	-0.65	**C12orf30**	chromosome 12 open reading frame 30	AW511198	chr12q24.13
1552612_at	-0.65	**CDC42SE2**	CDC42 small effector 2	NM_020240	chr5q31.1
212574_x_at	-0.65	**C19orf6**	chromosome 19 open reading frame 6	AC004528	chr19p13.3
1564931_at	-0.65	**---**	---	AL512740	---
1557918_s_at	-0.64	**SLC16A1**	solute carrier family 16, member 1 (monocarboxylic acid transporter 1)	AU131482	chr1p12
1555609_a_at	-0.64	**ZMAT3**	zinc finger, matrin type 3	AF355465	chr3q26.3-q27
205446_s_at	-0.64	**ATF2**	activating transcription factor 2	NM_001880	chr2q32

212307_s_at	-0.64	**OGT**	O-linked N-acetylglucosamine (GlcNAc) transferase	BF001665	chrXq13
1557071_s_at	-0.64	**NUB1**	negative regulator of ubiquitin-like proteins 1	BQ272330	chr7q36
213872_at	-0.63	**---**	---	BE465032	---
214483_s_at	-0.63	**ARFIP1**	ADP-ribosylation factor interacting protein 1	AF124489	chr4q31.3
1558093_s_at	-0.63	**MATR3**	matrin 3	BI832461	chr5q31.2
213807_x_at	-0.63	**MET**	met proto-oncogene (hepatocyte growth factor receptor)	BE870509	chr7q31
222145_at	-0.63	**---**	---	AK027225	---
1563629_a_at	-0.62	**---**	---	AK091794	---
1556035_s_at	-0.62	**ZNF207**	zinc finger protein 207	AI201248	chr17q11.2
1553105_s_at	-0.62	**DSG2**	desmoglein 2	NM_001943	chr18q12.1
231274_s_at	-0.62	**---**	---	R92925	---
207564_x_at	-0.61	**OGT**	O-linked N-acetylglucosamine (GlcNAc) transferase	NM_003605	chrXq13
1563894_at	-0.61	**LOC441178**	hypothetical LOC441178	AK056600	chr6q27
1555434_a_at	-0.61	**SLC39A14**	solute carrier family 39 (zinc transporter), member 14	BC015770	chr8p21.3
1555167_s_at	-0.61	**NAMPT**	nicotinamide phosphoribosyltransferase	BC020691	chr7q22.2
229524_at	-0.61	**KIAA0494**	KIAA0494	AI040880	chr1pter-p22.1
1553613_s_at	-0.61	**FOXC1**	forkhead box C1	NM_001453	chr6p25
235150_at	-0.60	**---**	---	AW376955	---
212193_s_at	-0.60	**LARP1**	La ribonucleoprotein domain family, member 1	BE881529	chr5q33.2
206788_s_at	-0.60	**CBFB**	core-binding factor, beta subunit	AF294326	chr16q22.1
232490_s_at	-0.60	**PRUNE**	prune homolog (Drosophila)	U67085	chr1q21
238443_at	-0.60	**---**	---	BE546873	---
1558249_s_at	-0.60	**STX16**	syntaxin 16	BE878126	chr20q13.32
1553685_s_at	-0.60	**SP1**	Sp1 transcription factor	NM_138473	chr12q13.1
1556416_s_at	-0.60	**---**	---	AF086242	---
1555326_a_at	-0.60	**ADAM9**	ADAM metallopeptidase domain 9 (meltrin gamma)	AF495383	chr8p11.23
210655_s_at	-0.60	**FOXO3**	forkhead box O3	AF041336	chr6q21
201211_s_at	-0.60	**DDX3X**	DEAD (Asp-Glu-Ala-Asp) box polypeptide 3, X-linked	AF061337	chrXp11.3-p11.23
1555639_a_at	-0.59	**RBM14**	RNA binding motif protein 14	AF315633	chr11q13.1
1552813_at	-0.59	**KLF14**	Kruppel-like factor 14	NM_138693	chr7q32.2
242824_at	-0.59	**---**	---	AW191647	---
212546_s_at	-0.59	**FRYL**	FRY-like	AI126634	chr4p12
224852_at	-0.59	**TTC17**	tetratricopeptide repeat domain 17	BE964325	chr11p12-p11.2
235219_at	0.59	**LOC116349**	hypothetical protein BC014011	BE897119	chr5p15.33
241912_at	0.59	**LOC730051**	similar to hCG2044798	AA631812	chr19q13.43
219493_at	0.59	**SHCBP1**	SHC SH2-domain binding protein 1	NM_024745	chr16q11.2
228092_at	0.59	**CREM**	cAMP responsive element modulator	AL552470	chr10p11.21
1559848_at	0.59	**NSUN4**	NOL1/NOP2/Sun domain family, member 4	BC016907	chr1p34
231183_s_at	0.59	**JAG1**	Jagged 1 (Alagille syndrome)	AI457817	chr20p12.1-p11.23
1557383_a_at	0.59	**---**	---	AI925316	---
217520_x_at	0.60	**LOC731884**	Similar to programmed cell death 6 interacting protein	BG396614	---

208297_s_at	0.60	**EVI5**	ecotropic viral integration site 5	NM_005665	chr1p22.1
210127_at	0.60	**RAB6B**	RAB6B, member RAS oncogene family	BC002510	chr3q22.1
211673_s_at	0.60	**MOCS1**	molybdenum cofactor synthesis 1	AF034374	chr6p21.3
227092_at	0.60	**---**	---	AI341383	---
1554057_at	0.60	**LOC645676**	hypothetical LOC645676	BC017347	chr1q22
234995_at	0.60	**CCDC52**	coiled-coil domain containing 52	AA668779	chr3q13.2
236076_at	0.60	**LOC257396**	hypothetical protein LOC257396	AW241549	chr5q11.2
201163_s_at	0.61	**IGFBP7**	insulin-like growth factor binding protein 7	NM_001553	chr4q12
226533_at	0.61	**HINT3**	histidine triad nucleotide binding protein 3	BF694956	chr6q22.32
240240_at	0.61	**---**	---	R10087	---
227535_at	0.62	**C15orf24**	Chromosome 15 open reading frame 24	AA149655	chr15q14
232541_at	0.62	**---**	---	AK000106	---
236752_at	0.63	**---**	---	AA913146	---
238931_at	0.63	**METT10D**	methyltransferase 10 domain containing	AL045793	chr17p13.3
219531_at	0.63	**CEP72**	centrosomal protein 72kDa	NM_018140	chr5p15.33
228832_at	0.64	**FLJ20021**	hypothetical LOC90024	AI884670	chr4q23
228737_at	0.64	**TOX2**	TOX high mobility group box family member 2	AA211909	chr20q13.12
201171_at	0.65	**ATP6V0E1**	ATPase, H+ transporting, lysosomal 9kDa, V0 subunit e1	NM_003945	chr5q35.2
212490_at	0.65	**DNAJC8**	DnaJ (Hsp40) homolog, subfamily C, member 8	AA843895	chr1p35.3
221530_s_at	0.66	**BHLHB3**	basic helix-loop-helix domain containing, class B, 3	BE857425	chr12p11.23-p12.1
1565484_x_at	0.67	**EGFR**	epidermal growth factor receptor	AF277897	chr7p12
225536_at	0.67	**TMEM54**	transmembrane protein 54	AL545105	chr1p35-p34
239022_at	0.67	**SDHALP1**	succinate dehydrogenase complex, subunit A, flavoprotein pseudogene 1	AW090199	chr3q29
239154_at	0.68	**---**	---	AW962705	---
213704_at	0.68	**RABGGTB**	Rab geranylgeranyltransferase, beta subunit	AA129753	chr1p31
1554478_a_at	0.70	**HEATR3**	HEAT repeat containing 3	BC033077	chr16q12.1
226675_s_at	0.70	**MALAT1**	metastasis associated lung adenocarcinoma transcript 1 (non-protein coding)	W80468	chr11q13.1
209603_at	0.73	**GATA3**	GATA binding protein 3	AI796169	chr10p15
221911_at	0.73	**ETV1**	ets variant gene 1	BE881590	chr7p21.3
1561347_a_at	0.74	**---**	---	AW303888	---
217551_at	0.75	**LOC441453**	similar to olfactory receptor, family 7, subfamily A, member 17	AA719797	chr11p15.1
236358_at	0.78	**---**	---	BF445865	---
226765_at	0.82	**SPTBN1**	Spectrin, beta, non-erythrocytic 1	AA971514	chr2p21
36711_at	0.85	**MAFF**	v-maf musculoaponeurotic fibrosarcoma oncogene homolog F (avian)	AL021977	chr22q13.1
1568838_at	0.85	**LOC100128[illegible]**	similar to hCG1742852 /// similar to hCG1742852	AI015847	chr16p13.3
213550_s_at	0.87	**TMCO6**	transmembrane and coiled-coil domains 6	AA993683	chr5q31.3
225767_at	0.93	**LOC284801**	hypothetical protein LOC284801	AI825833	chr20p11.1
228697_at	1.12	**HINT3**	histidine triad nucleotide binding protein 3	AW731710	chr6q22.32
244780_at	1.24	**SGPP2**	sphingosine-1-phosphate phosphotase 2	AI800110	chr2q36.1
